# Measuring teacher self-efficacy: Validating a new comprehensive scale among Chinese pre-service teachers

**DOI:** 10.3389/fpsyg.2022.1063830

**Published:** 2023-01-24

**Authors:** Kang Ma, Jiutong Luo, Michael Cavanagh, Jingjing Dong, Meng Sun

**Affiliations:** ^1^Jiangsu Provincial Key Constructive Laboratory for Big Data of Psychology and Cognitive Science, Yancheng Teachers University, Yancheng, Jiangsu, China; ^2^Advanced Innovation Center for Future Education, Faculty of Education, Beijing Normal University, Beijing, China; ^3^School of Education, Macquarie University, Sydney, NSW, Australia; ^4^College of Education for the Future, Beijing Normal University, Zhuhai, Guangdong, China

**Keywords:** teacher self-efficacy, student teachers, teacher education, scale development, validation

## Abstract

Challenges exist in the validating procedure and comprehensiveness of the existing TSE measurements, though advancements have been achieved. Also, less consistencies have been received regarding teacher self-efficacy measurement in Chinese context so that the study developed and validated a new comprehensive scale for this construct. A total of 854 Chinese pre-service teachers responded to 40 purposely selected teacher self-efficacy items, together with the Generalized Self-Efficacy Scale, the agreeableness subscale of the Big Five Inventory, and items on their effectiveness of teaching practicing and intention to be a teacher. Exploratory factor analyses revealed two distinct factors, one factor (Ethos) focused on the general school climate, harmony, and cooperation, as well as teachers’ own professional development, the other (Teaching) focused on aspects of classrooms and student learning. Confirmatory and second-order factor analysis supported the existence of two factors and also indicated one overarching construct of teacher self-efficacy. Both domains were significantly correlated with general self-efficacy and agreeableness, with either moderate or low correlations. Significant differences in teacher self-efficacy for Ethos and Teaching were found between pre-service teachers who reported higher levels of effectiveness during their professional placement and greater intention to be teachers compared to those with lower self-ratings. In addition, a 20-item short version of the scale was developed, and the same factorial structure was confirmed. This study validated the two-factor structure of a newly developed teacher self-efficacy scale that covers domains both within and outside classroom teaching. Limitations and implications are discussed.

## Introduction

In recent years there has been a growing interest in research on teacher self-efficacy (TSE), defined as an individual teacher’s perception of their capability to accomplish the tasks required to be an effective teacher (Kleinsasser, 2014; Mok and Moore, 2019; Pawlak, 2022; Wyatt, 2022). TSE has been associated with improvements in teacher effectiveness (Boulden et al., 2021; Noorollahi, 2021) and reductions in teacher attrition (Klassen and Tze, 2014). TSE could also significantly predict students’ learning motivation, learning enjoyment and academic performance across varying school environments (Fackler and Malmberg, 2016; Hettinger et al., 2021).

Research in this field has shown significant advancements, including in the measurement of TSE; however, certain methodological limitations exist (see Zee and Koomen, 2016; Ma et al., 2021b). One predominant characteristic of the commonly applied TSE scales (e.g., Teacher Sense of Efficacy Scale [TSES], Tschannen-Moran and Hoy, 2001) has been a focus on classroom teaching (Bin Khairani and Bin Abd Razak, 2012). Such measures might be incapable of covering all essential domains of being a teacher (Avanzi et al., 2013; Karbasi and Samani, 2016) or even teaching. For example, integrating technology into teaching (Gomez et al., 2022) and enhancing democratic cooperation among students (Wheatley, 2005), which could truncate TSE’s predictability (Bandura, 2012). Recent research has also identified certain deficiencies in validating procedures used in the most commonly applied TSES. For instance, the usage of orthogonal rotation when the subdomains were correlated (Ma et al., 2020) and reliance on eigenvalues and scree plots instead of methods like parallel analysis that could elicit more accurate estimation of the number of factors (Lim and Jahng, 2019). These might have inflated the number of factors, which has advocated for rigorous research on TSE measurement (Koniewski, 2019; Salas-Rodríguez et al., 2021). Furthermore, the call for research from a more international perspective has been made (Klassen and Durksen, 2014). Research in the measurement of TSE in the Chinese context is limited (Ma et al., 2020; Yin et al., 2020); therefore, the current study aims to validate a comprehensive TSE scale among Chinese pre-service teachers, incorporating recent advancements in TSE measurement.

## Teacher self-efficacy: Theory and measurement

Although Bandura’s social cognitive theory has guided research on TSE (Pfitzner-Eden, 2016), questions have been raised about the theoretical accuracy, item identification, and specificity of TSE measurement (Chesnut and Burley, 2015; Kim et al., 2019).

Self-efficacy beliefs reflect an individual’s future-oriented anticipation of their capability to accomplish specific tasks (Bandura, 1993). It differs from constructs like confidence, with the latter being a generalized trait and the former being context-specific (Bandura, 2012). The wording or expression of self-efficacy items, therefore, should be specific regarding the tasks to be accomplished and include wording such as “able” and “capable” (Bandura, 2012). However, it is common for items in TSE measures to contain wording like “I feel confident” (Pfitzner-Eden, 2016), which unnecessarily conflate the differing concept of confidence with the “can do” notion of capability (Bandura, 2006). Meanwhile, the necessity to differentiate self-efficacy and outcome expectancy has been raised with the latter being less predictive in human behaviors (Bandura, 2012). Concerns have been raised about the predictability of TSE items without considering beliefs in the effectiveness of such behaviors (Kim and Kim, 2010; Chesnut and Burley, 2015) as individuals might not be motivated without considering individual beliefs about the outcome (Bai et al., 2022). However, such opinions might have failed to resonate with the suggestions of Bandura (2012) to not confound the measure of self-efficacy with related effects.

The most commonly applied TSE scale is the TSES (Tschannen-Moran and Hoy, 2001), which emphasises the measurement of TSE in three classroom teaching domains: maintaining classroom discipline, involving students in teaching, and employing various instructional strategies (Perera et al., 2019). The domain specificity was intended to increase the predictability of the construct and increase the practical applicability of research findings to the specific contexts of teachers’ work (Avanzi et al., 2013). However, individuals’ perception of their capability varies across different domains and dimensions within each domain (Yang et al., 2022); hence, if TSE is measured in too narrow a domain, educators may be deterred from applying it in practice (Bandura, 2012). As noted by Bandura (2012), individuals tend to evaluate their self-efficacy towards specific tasks simultaneously with an assessment of their general ability. For example, teachers might hold a broader general view of their capability as a teacher in the school context, alongside their self-efficacy for specific tasks. These theoretical assumptions have been confirmed by variations in TSE for subscales and the integrated information on the higher-order overall TSE (Perera et al., 2019).

Much evidence on the three-domain TSE structure, mainly based on the TSES, has been confirmed (Valls et al., 2020); further inspections appear to be needed. On the one hand, regarding the statistical techniques, the orthogonal rotation has been utilized, assuming factors were uncorrelated, in factor structure studies, whereas the correlations between TSES subscales range from 0.58 to 0.70 and from 0.95 to 0.98 for the long and short formats, respectively. This rotation method may have enlarged the number of factors retained (see Burgueño et al., 2019; Perera et al., 2019; Ma et al., 2020). The correlations between these subdomains, calculated in subsequent studies, average to be larger than 0.6 (Koniewski, 2019; Salas-Rodríguez et al., 2021). Then, both eigenvalues and scree plots have been predominantly applied to decide the number of factors; however, parallel analysis has been demonstrated to outperform these traditional methods in producing a more accurate factorial structure (Preacher and MacCallum, 2003). Such an advantage of parallel analysis was based on comparing the eigenvalues of the sample with those received from a random correlation matrix without assuming preliminary factor numbers (Lim and Jahng, 2019). However, such a method has rarely been applied in TSE research since the call was made over two decades ago (Henson, 2002). Furthermore, none of the validation studies of the TSES reviewed by Koniewski (2019) reported sufficient chi-square and the average of RMSEA was 0.077 [0.053, 0.134], which slightly exceeded the cut-off values. On the other hand, in terms of research involving pre-service teachers (PSTs), the use of one overall factor has been recommended (Tschannen-Moran and Hoy, 2001) as there is insufficient evidence of the factor structure being relevant in this population, yet the three-domain structure has been applied among PSTs (e.g., Burgueño et al., 2019). The failure to confirm the three-factor structure could explain findings of PSTs’ incapability to comprehend teaching complexity (Fives and Buehl, 2009; Duffin et al., 2012).

Nonetheless, questions have emerged about the nature and assessment of TSE in domains beyond the classroom walls. Cherniss (1993) proposed a framework combining teachers’ capabilities to perform instruction, disciplining, and student engagement within the classroom, establishing interpersonal connections with other professionals and making an impact on the function of the school. Friedman and Kass (2002) commented that domains other than classroom teaching are “undisclosed” and include the ability to socialize with school staff and students, with similar findings in a study conducted by Skaalvik and Skaalvik (2007). Bandura (2006) initiated seven dimensions of TSE: influencing school administrative decisions, acquiring school resources, instruction, discipline maintenance, engaging parents, involvement with communities, and building a positive school climate. Including domains other than classroom teaching is especially meaningful as, according to Bandura, TSE measurements should be “broad in scope and domain specificity” (2012, p. 17). It is also essential to emphasize such a line of research in initial teacher education, because using TSE scales with a narrow scope on classroom teaching not only fails to ensure the predictability of TSE, but is also not able to examine PSTs’ TSE for tasks other than classroom teaching (Cocca and Cocca, 2021), which were reported to be the main reason for ‘reality shock’ once PSTs transfer to their first year of teaching (Ma et al., 2021a).

## Teacher self-efficacy measurement in the Chinese context and the present study

Chinese teachers, including PSTs, have been found to have a perception of teaching responsibilities differing from their western counterparts; for instance, their preferences for teacher-oriented instruction, exercising parental authenticity, and ethnical care for students (Lin and Gorrell, 2009; Liu et al., 2022). Such differences might be rooted in the Chinese paternalism perspective of teacher morality that addresses teacher authority and moral guidance, together with teaching competence (Ho and Hau, 2004; Ye and Zhou, 2020; Kutuk et al., 2022). Consider the Chinese teacher creed, for instance, Great learning makes a teacher and moral integrity makes a model. Chinese PSTs have easier access to parental assistance than PSTs in the US and do not consider receiving help from parents indicates lower TSE (Cheung, 2006). Meanwhile, Chinese teachers influenced by collectivistic cultures tend not to be as positive about their teaching capability, compared to those from individualistic backgrounds (Vieluf et al., 2013), which has been consistently reported in Asian countries (Ruan et al., 2015). However, the likelihood of teachers to rate their TSE lower in collectivist countries, especially in Asia, could, paradoxically, exhibit more effective teaching strategies in their practice (Vieluf et al., 2013).

Research in the Chinese context has either developed TSE scales initially based on teachers’ responsibilities or they have been adapted from an existing scale (e.g., TSES). In a Hong Kong sample, Chan (2008a) selected 24 items to measure six pre-determined domains: teaching highly capable students, classroom management, providing guidance and counselling, student engagement, teaching in diversified contexts, and teaching for creativity, critical thinking, and problem-oriented teaching. However, the original model fit for confirmatory factor analysis (CFA) was not satisfactory, before the number of items was reduced to 18. In a sequential study, Chan (2008b) added three items to capture interactions with colleagues and parents to make a seven-domain scale, despite the recommendation that there should be at least four items per factor (Fabrigar et al., 1999). It appears unclear whether the seven factors could converge to a high-order factor, due to the high correlations between the different factors of guidance and counselling and student engagement (Chan, 2008b). In both studies, data from both in-service teachers (ISTs) and PSTs were combined, despite the possibility that the factor structure might differ for each.

The TSES does not appear to elicit a consistent factor structure in the Chinese context. Cheung (2008) found one overall factorial structure among the whole sample and confirmed a two-factorial structure among the male group, namely efficacy for student engagement and efficacy for student disciplining. The two factors consisted of items mixed from different original TSES factors. Tsui and Kennedy (2009) confirmed a two-factor structure, efficacy in teaching and assisting students’ learning, by mixing items for instructional strategies, student engagement, and classroom management. Ruan et al. (2015) validated the TSES in China, Japan, and Korea, and found the 24-item version failed to produce a satisfactory model fit and the 12-item version only received an acceptable model fit after being reduced to 11 items, with modification index fixation being applied. Ma et al. (2020) confirmed a unified one-factorial structure among in-service and PSTs, after making adaptations to item wording and response options; however, we raised concerns that the short form of the TSES might be limited in domain breath.

Many improvements, as noted above, have been achieved in TSE measurement, however, certain challenges exist including the validation procedure and domain comprehensiveness. The latter is especially essential considering the wholistic view of teacher professional development which included tasks both within and external classroom teaching. Furthermore, limited agreements have been achieved in validating a solid TSE measurement, especially considering the inconsistencies in the factorial structure of TSES, in Chinese context. Therefore, this study aims to develop a TSE scale to use with Chinese PSTs by incorporating factor analysis methodological recommendations and broader domains of teaching.

## Materials and methods

### TSE item pool

The item pool, with 40 items, was generated from a range of sources. Initially, 43 items were selected for inclusion. Six items were selected from the TSES short form that carried moderate, rather than strong, correlations with each other in our prior research; for example, *Getting students to believe they can do well at school* (Ma et al., 2020). We added six items from the long form to broaden the construct (e.g., *Providing appropriate challenges for very capable students*). An additional 31 items were generated by referring to other publications (e.g., Bandura, 2006; Zee and Koomen, 2016). Among the 31 items, nine were similar to the TSES items and explicitly focused on classroom teaching (e.g., *Monitoring students’ focus while you are teaching*; see Dellinger et al., 2008), with five of these items covering integrating technology in teaching (e.g., *Capturing students’ interest through your use of technology*; see Wang et al., 2004; Mayo et al., 2005; Sang et al., 2010). We also employed three items about motivating students to conduct peer cooperation (e.g., *Encouraging students to take on a leadership role*; see Wheatley, 2005; Dellinger et al., 2008), three items about enhancing teacher-student and student–student interactions (e.g., *Establishing positive and enjoyable interactions between you and your students*; see Friedman and Kass, 2002), 13 items focusing on involvement with colleagues, the school administration and other stakeholders, including parents and the community (e.g., *Maintain effective communication with the school principal;* see Cherniss, 1993; Friedman and Kass, 2002; Skaalvik and Skaalvik, 2007) and three items that dealt with intrapersonal aspects, namely reacting effectively to educational reforms, maximizing professional development opportunities, and balancing pressures from both life and work (e.g., *Coping with challenges brought about by changes in the educational environment*; see Friedman and Kass, 2002; Skaalvik and Skaalvik, 2007; Zee and Koomen, 2016). Although we used the above categories as a means of guiding the generation of items, we did not assume these categories would comprise distinct clusters, for example, in subsequent factor analyses.

A process of relevance assessment was conducted in a preliminary analysis with 25 ISTs in China, who were from preschools (*n* = 7), primary schools (*n* = 7), junior high schools (*n* = 6), and senior high schools (*n* = 5). The relevance was rated based on a 9-point response with 1, 3, 5, 7 being labeled as *minimally relevant, only moderately relevant, quite relevant and extremely relevant*, and 8 and 9 being rated as *I really have no idea at all* and *not applicable*. The relevance was tested based on content validity ratios (CVRs). Nine items (e.g., *Keeping good records for your school and for regulatory authorities*) had CVRs <0.28 and five of them appeared to be adequately represented by other items among the 43 items. We therefore discarded them according to recommendations by Wilson et al. (2012) that CVRs ≥0.392, when 25 participants rated their relevance, and CVRs ranging up to ≥0.428, when as few as 21 participants provided relevance ratings. In addition, two items (*Dealing with administrative tasks* and *Helping students to enjoy being at school*) that were overlooked were added. Therefore, the final scale contained 40 items. Participants were invited to *indicate how effective you think you will be in each of the following activities* with responses on the 9-point response scale with 1, 3, 5, 7, 8 and 9 being *labeled minimally effective, only moderately effective, quite effective, extremely effective, I really have no idea at all and not applicable* and 2, 4, and 6 not labelled. Such a method of labelling, as used by Skaalvik and Skaalvik (2007) outperformed only labelling the extreme responses in producing a higher quality of data and psychometric outcomes in our preliminary experimental research (Trevethan and Ma, 2021). In addition, the last two options were added due to PSTs’ potential incapability with understanding certain items especially those without any teaching experience and the purpose to detect any potential items that might be irrelevant (see Duffin et al., 2012; Ma et al., 2020).

### Convergent and discriminant validation

A general sense of efficacy scale was administered for the purpose of assessing convergent validity, as self-efficacy has been found to correlate with TSE (Pfitzner-Eden et al., 2014). The 10-item Generalized Self-Efficacy Scale (GSE) is a single-factor scale, with Cronbach’s alpha ranging from 0.75 to 0.90 across 23 nations (Schwarzer and Jerusalem, 1995). When validated with a Chinese sample of students, the scale had a single factor and an alpha of 0.91 (Zhang and Schwarzer, 1995). We altered the response options to a 7-point scale, with 1, 3, 5, and 7 labeled as *never, sometimes*, *often and always*. Cronbach’s alpha in the present study is 0.958.

The agreeableness scale of the Big-Five Personality Scale has been found to have a low association with TSE among PSTs (Senler and Sungur-Vural, 2013) and ISTs (Bullock et al., 2015) and was, thus, applied to measure discriminant validity. The 9-item agreeableness subscale of the Big Five Inventory has a Cronbach’s alpha higher than 0.83 in studies (John and Srivastava, 1999). This scale comprises a single factor for both adolescents and adults in China and has a Cronbach’s alpha of 0.80 (Meng et al., 2021). We applied a 7-point response scale, with 1, 3, 5, and 7 labeled as *never*, *sometimes*, *often and always*. Cronbach’s alpha in the present study is 0.775.

### Known-groups validation

Two single-topic questions were employed to test known-groups validation. First, PSTs who reported their teaching practice as effective were compared to those who regarded it as ineffective. This question was selected because PSTs who experience failures during their practicum reported lower TSE (Martins et al., 2015). Second, PSTs who had a strong desire to become teachers after completion of their course were compared with those who were less motivated to be teachers. This was based on prior research showing highly efficacious PSTs tend to be more committed to (Chesnut and Burley, 2015; Zee and Koomen, 2016), and become more optimistic about their career (McLennan et al., 2017).

### Participants

An online survey invitation was administered *via* social media to 984 PSTs from two teacher education institutions in China, one a city-level college and the other a provincial university. A total of 854 PSTs provided analyzable data, with data excluded from participants selecting response options 8 and 9, and those providing identical responses on the TSE items (*n* = 130, 13.2%). Demographic details are presented in Table 1. Eighty-one percent of respondents were in either the second or third year of their studies, and slightly over 90% were in either a 3-year diploma or 4-year bachelor course and were 21 or 22 years of age. The majority of respondents were female (95.1%). A minority of PSTs (17.3%) indicated they had not yet been on practicum placements, but nearly half (41.8%) had already been on placements during which they believed they had been either highly or very highly effective. Nearly half of respondents (44.8%) indicated they definitely wanted to become teachers after graduating.

**Table 1:** Background characteristics of participants (*N* = 854)^a^.

Variable	Number	%
**Year of study**		
First	52	6.1
Second	338	39.6
Third	347	40.6
Fourth	109	12.8
**Course**		
Three-year diploma	370	43.3
Four-year bachelor	414	48.5
A bachelor degree with prior diploma	64	7.5
**Age**		
<20	57	6.7
20–21	751	88.8
>22	38	4.4
**Sex**		
Female	802	95.1
Male	41	4.8
**Sense of effectiveness on practicum(s)**		
Very high	45	5.3
High	312	36.5
Moderate	327	38.3
Poor	10	1.1
Not applicable	148	17.3
**Intention to become a teacher after graduation**		
Definitely yes	383	44.8
Probably	273	32.0
Not sure	148	17.3
Probably not	33	3.9
Definitely not	6	0.7

^a^Percentages did not always add up to 100% due to missing data.

### Analyses

Data were analyzed using SPSS Version 22 and Mplus Version 8.1. The participants first were randomly separated into two groups with 427 PSTs in each so that EFAs and CFAs could be conducted with distinctive datasets. Second, the inter-item correlations, existence of multicollinearity, normality and outliers, together with KMO index and Bartlett’s test of sphericity were examined before examination of factor structure based on eigenvalues greater than 1, scree plot and parallel analysis. Maximum likelihood extraction and promax rotations were used for EFAs, in which cases with missing data were excluded pairwise.

Third, two sets of two-factor CFA with data from the second group of PSTs (*n* = 427) were conducted using the Maximum likelihood estimation. The first was conducted with the original 40 items and the second comprised the 10 highest-loading items on each factor. This decision was mainly based on the necessity to develop a short scale for users’ convenience, as in prior studies (e.g., retaining the first half of the highest loaded items in the TSES; Tschannen-Moran and Hoy, 2001).

Model fits of CFAs were evaluated based on the Chi-square (*χ^2^*) statistic, RMSEA, standardized root mean square residual (SRMR), comparative fit index (CFI) and Tucker-Lewis index (TLI). The cutoff values were chosen based on methodological studies (Hu and Bentler, 1999; Hooper et al., 2008; Boateng et al., 2018). Specifically, *χ^2^*/*df*, <3 being preferable, 3–5 being acceptable. For CFI and TLI, >0.95 being preferable, 0.90–0.95 being acceptable; for RMSEA and SRMR, <0.05 being preferable and 0.05–0.08 being acceptable. In addition, missing values were modelled using the full information maximum likelihood (FIML). Sequentially, reliabilities of the scales were calculated using Cronbach’s alpha, Composite reliability and McDonald’s Omega simultaneously (Kalkbrenner, 2021).

Finally, the convergent and discriminant validity and known-group validity were tested using data from 767 (78.2%) and 843 (86.7%) of the 984 PSTs with usable TSE data, respectively. Spearman’s rank-order correlations were calculated, as this is likely to yield more valid results than Pearson’s product–moment correlations (Bishara and Hittner, 2012). Also, the averaged variance extracted was calculated for convergent validity.^1^ Independent sample t-tests were conducted to examine the known-group validity.

## Results

### Exploratory and confirmatory factor analyses

The majority of inter-item correlations (*n* = 678, 86.92%) were not less than 50, none of which was higher than 0.90 with the average correlation being 0.62. No multicollinearity or outliers were detected and the KMO index (0.98) and Bartlett’s test of sphericity (*p* < 0.001) was satisfactory. In the preliminary analyses, four eigenvalues were larger than 1, and a sharp decline between the first and the rest eigenvalues, from 24.18 to 2.49, 1.40, 1.00, and 0.90. Parallel analysis indicated two factors using principal components and 1,000 randomly generated matrices. Thus, the EFA was continued with two factors using maximum likelihood extraction and promax rotation.

In this analysis, extraction commonalities were between 0.47 and 0.76 (*M* = 0.65, *SD* = 0.07), and the variance was explained by the two factors was 60.44 and 6.22%, respectively. The two factors that emerged were distinct, each with 20 items, and no cross-loadings under 0.30 were evident. After examining the items within each factor, the two factors were labelled Ethos and Teaching factors. Specifically, Ethos covers items on general school climate, harmonious interaction among fellow teachers and students, and personal professional development – all of which reflect a holistic perspective of teaching as a profession. The Teaching factor covers items relating to general classroom teaching and student learning. The item loadings for Ethos ranged from 0.57 to 0.93, and for Teaching, from 0.53 to 0.85 (see Table 2), which had an inter-factor correlation of 0.78.

**Table 2:** Factor loadings in EFA (*N* = 427).

Item^a^	Factor1	Factor2	Version^b^
Contributing to a positive school climate	0.93		S
Using opportunities for your own professional development	0.92		S
Gaining support for your school from parents	0.92		S
Gaining support for school development in your area	0.90		S
Dealing with work pressure along with pressure from other sources, such as family and society	0.89		S
Being respected by colleagues, the principal, and others	0.88		S
Expressing your views freely about important school matters	0.87		S
Obtaining resources when you need them	0.86		S
Helping your students perform better compared with other students under the same conditions	0.85		S
Obtaining assistance from colleagues	0.77		S
Working with colleagues to maximize use of technology in teaching	0.75		L
Coping with challenges brought about by changes in the educational environment	0.74		L
Providing assistance to colleagues	0.72		L
Guiding and counseling students	0.71		L
Establishing positive and enjoyable interactions between you and your students	0.66		L
*Getting students to follow classroom rules*	0.66		L
*Making your expectations about student behavior clear*	0.65		L
Gaining respect from students	0.62		L
*Gaining support from parents with regard to their children’s learning*	0.57		L
*Redirecting students who are persistently off task*	0.57		L
*Getting students to believe they can do well at school*		0.85	S
Providing appropriate guidance for students who have learning difficulties		0.83	S
*Gauging student comprehension of what you have taught*		0.79	S
*Providing appropriate challenges for very capable students*		0.78	S
Capturing students’ interest through your use of technology		0.78	S
Monitoring students’ focus while you are teaching		0.76	S
*Providing an alternative explanation or example when students are confused*		0.74	S
*Helping students to value learning*		0.73	S
Integrating computers and other technology in your teaching		0.73	S
Encouraging students to be cooperative with each other		0.68	S
Planning and preparing for high quality teaching		0.67	L
Assessing students’ work		0.67	L
Encouraging students to respect each other		0.65	L
*Using a variety of assessment strategies*		0.64	L
Encouraging students to take on leadership roles		0.64	L
*Encouraging students to examine and solve problems independently*		0.58	L
Keeping well-maintained records of student performance		0.57	L
*Helping students to think in unconventional, creative, and productive ways*		0.57	L
Dealing with administrative tasks		0.54	L
Helping students to enjoy being at school		0.53	L
Eigenvalue	24.18	2.49	
% of Total variance	60.44	6.22	
Total variance	66.66%		

^a^Italicized items were from the TSES.^b^S indicates items included in the short version (also belong to the long version) and L indicates items only included in the long scale.

Two sets of CFAs were conducted and simultaneously analyzed with the correspondent second-order factor analysis, considering the high inter-factor correlation (*r* = 0.86 and 0.741, respectively). The model fits of all four models were satisfactory other than TLI for the two original two-factor models were slightly lower than 0.90. For all four models, *χ^2^*/*df* ranged from 2.265 to 3.602, CFI ranged from 0.903 to 0.944, TLI ranged from 0.896 to 0.940, RMSEA ranged from 0.073 to 0.078 and SRMR ranged from 0.041 to 0.046 (see Table 3). It is noteworthy that the errors of five pairs of items, each of two being in sequential order, were allowed to be correlated in both the original two-factor CFA and the second-order analysis to improve the model fits. Such a decision was assumed to be reasonable because the error correlation could be allowed when either theoretical or methodological reasons could be assumed (Brown, 2015; Smolkowski, 2020) and in the current study, the larger number of items might have increased the cognitive burden of participants. Besides, the two versions of scale received satisfactory reliabilities. Regarding the Cronbach’s alpha, it was 0.97 and 0.95 for Ethos, and 0.98 and 0.96 for Teaching, correspondingly in the long and short scales. Regarding the composite reliability, it was 0.67 and 0.80 for Ethos, and 0.57 and 0.65 for Teaching, respectively. The McDonald’s Omega reliability reached 0.979 and 0.962 for Ethos, and 0.970 and 0.946 for Teaching in the two versions of scales correspondingly.

**Table 3:** Results of four CFAs (*n* = 427).

Model	*χ^2^*	*df*	*p*	*χ2/df*	CFI	TLI	RMSEA	SRMR
Model 1^a^	2417.586	729	<0.001	3.316	0.903	0.896	0.074	0.043
Model 2^b^	608.69	169	<0.001	3.602	0.944	0.937	0.078	0.041
Model 3^c^	2438.854	747	<0.001	3.265	0.903	0.898	0.073	0.046
Model 4^d^	613.767	177	<0.001	3.468	0.944	0.940	0.076	0.042

^a^CFA for the original two-factor structure; ^b^CFA for two-factor structure with 10 items with highest loadings in each factor and labeled with S in Table 2; ^c^Second-order analysis for model 1; ^d^Second-order analysis for model 2.

### Convergent and discriminant validity

As to convergent validity, a moderate statistically significant correlation was found between GSE and TSE for Ethos and Teaching, *r* (752) = 0.41, *p* < 0.001and *r* (741) = 0.39, *p* < 0.001, respectively. Meanwhile, the averaged variance extracted (AVE) reached 0.61 for Ethos and 0.48 for Teaching^2^. Regarding the discriminant validity, a low but statistically significant correlation was examined between agreeableness and TSE for Ethos and Teaching, *r* (741) = 0.25, *p* < 0.001 and *r* (730) = 0.25, *p* < 0.001, respectively.

### Known-groups validity

A total of 355 and 484 PSTs reported themselves to be in the highest two categories of effectiveness and lowest three categories, respectively. Regarding Ethos, the difference between the former (*M* = 4.7, *SD* = 1.30) and latter (*M* = 4.36, *SD =* 1.32) groups was significant, *t*(837) = 3.735, *p* < 0.001. For Teaching, the difference between the former (*M* = 4.65, *SD* = 1.22) and latter (*M* = 0.31, *SD* = 1.25) groups was significant, *t*(839) = 3.883, *p* < 0.001.

A total of 383 (44.8%) and 470 (55.2%) PSTs indicated having the strongest and lowest intention to become teachers, respectively. Regarding Ethos, the difference between the former (*M* = 4.64, *SD* = 1.32) and latter (*M* = 4.4, *SD* = 1.31) groups was significant, *t*(849) = 2.638, *p* = 0.008. For Teaching, the difference between the former (*M* = 4.62, *SD* = 1.23) and latter (*M* = 4.32, *SD* = 1.25) groups was significant, *t*(851) = 3.532, *p* < 0.001.

## Discussion

The current study aimed to validate a comprehensive TSE scale using an initial 40 items among Chinese PSTs. It confirmed an overarching TSE factor with two subscales, named as Ethos and Teaching. The study also found significant moderate and low correlations between TSE and another two constructs, namely general self-efficacy and agreeableness, respectively. In addition, it found highly efficacious PSTs tended to report higher effectiveness regarding their professional experience and stronger intention to become teachers.

The new TSE scale had a two-factor structure, namely Ethos and Teaching. This finding is promising, following the call for a TSE scale that is broader and covers essential domains of being a teacher not only within the classroom but also within the school context (Wheatley, 2005; Cocca and Cocca, 2021). The existence of two factors, especially the Ethos factor, is supported by the low proportion of missing data and infrequent use of the *no idea* and *not applicable* response options, suggesting the high relevance of the 40 purposely selected items as revealed in the preliminary relevance rating. The distinct factorial structure could also have benefited by applying the response scale with labels on odd numbers, which was reported to produce better data quality and psychometric results by assisting PSTs to rate TSE more accurately (Trevethan and Ma, 2021). Meanwhile, this structure was supported because, first, two distinct factors were indicated in the parallel analysis; second, there was a totally distinct pattern of loadings on each domain in the EFA; third, the two-factor model fit in the CFA had a high degree of acceptability, and fourth, each factor comprised a meaningful cluster of items that, as noted above, corresponded with the two-factor solutions theorized and identified in other research (e.g., Friedman and Kass, 2002; Skaalvik and Skaalvik, 2007). However, to achieve the satisfied model fit indices in CFAs, this study allowed five pairs of neighboring items to be correlated. This might not be problematic, considering the large number of items increases the challenge of obtaining a satisfactory model fit in CFA (see Matsunaga, 2010; Smolkowski, 2020), particularly the root mean square error of approximation (RMSEA; Schermelleh-Engel et al., 2003). Especially, the model fits for both the two-factor structure and the second-order analysis, based on the 10 items with the highest loadings for each factor, were satisfactory without making any modifications. Such a short 20-item scale tapping broad domains of the teaching profession could be preferrable, considering the convenience it offers researchers. In addition, the model fits could have been more acceptable if extra items were removed, especially retaining only the five items with the highest factor loadings, which was the method used to extract the short form of the TSES (Tschannen-Moran and Hoy, 2001). However, by removing items to obtain more desirable goodness-of-fit indices, we would have jettisoned the main aim of this research, namely, to create a scale with wide content coverage, and we would have done so to satisfy goodness-of-fit rules of thumb that are increasingly being shown to have insubstantial foundations (Brown, 2015).

All items that were either from or bear similar meanings with those in the study of Bandura (2006) had sufficient factor loadings; however, they formed two broad factors with five domains external to classroom teaching, such as being involved with parents, integrated to be one holistic factor. The current factorial structure is more aligned with the personal and professional dimensional structure suggested by Friedman and Kass (2002). The two-factor structure, forming a higher-order structure, might have extended the seven-dimension structure of Chan (2008a,b), who failed to provide an examination of the higher-order structure that tapped domains related to both classroom teaching and activities at the school level. This is especially meaningful considering the high correlations between the two subdomains, namely student engagement and guidance and counselling students in the study of Chan (2008a,b), which might indicate the need to conduct a second-order factor analysis.

TSES items included in the present study divided into two factors. This also occurred with TSES data from ISTs in Hong Kong (Tsui and Kennedy, 2009) and Shanghai (Cheung, 2006), in which the TSES engagement and instruction items loaded on one factor (Teaching, in the current research) and the TSES management loaded items on the other (Ethos, in the current research). Of particular interest, items based on the TSES management factor were among the items with the lowest loadings on Ethos in our research, suggesting that participants in our study regarded management of students to be less salient than other aspects of teachers’ professional experiences and preoccupations. This pattern appears to confirm the finding that Chinese PSTs might have differentiated tasks other than classroom instruction with those associated with moral guidance, which might have given them more authority to correct students’ misbehaviors in, but not limited to, classroom teaching contexts (Ye and Zhou, 2020). This supports a need for considering cross-cultural differences in the foundations of TSE (see, for example, Ho and Hau, 2004).

The varying distributions of TSES items in the current study are also consistent with prior studies (Cheung, 2006) that confirmed a two-factor structure, namely, TSE for enhancing students’ learning and behavior management among Chinese participants. This is also consistent with Labone (2004) who addressed the necessity to extend the engagement domain within the classroom to broader social contexts, including parents and colleagues. Such a finding might be associated with a common understanding of being a teacher among Chinese teachers, including PSTs, that addresses the bond between teachers and students, such as the parenting and obedience relationship, as a separate domain than classroom teaching (Liu et al., 2022). This two-factor structure also aligns with another recent study that validated a revised TSES among both Chinese ISTs and PSTs that identified a one-factor structure, which, as assumed by the Ma et al. (2020), could be attributed to Chinese participants’ holistic view of teaching.

The correlations between general self-efficacy and the two subscales, Ethos and Teaching, were 0.41 and 0.39, lying within the span of 0.35 to 0.45 used by Pfitzner-Eden et al. (2014) to support convergent validity of their TSE scale. Meanwhile, the correlations of the two subscales of 0.25 with the agreeableness scale appeared to be higher than that reported in prior studies (e.g., |r| < 0.07 in Senler and Sungur-Vural, 2013; |*r*| < 0.14 in Bullock et al., 2015). Also, in absolute terms, these correlations are in the same region as the correlations of −0.31 and −0.21 claimed by Tschannen-Moran and Hoy (2001) as establishing discriminant validity for the TSES, in relation to work alienation and pupil control ideology. However, the influences of scales used for these different constructs on the correlation coefficients shall be taken into accounts. Further exploration of the research literature revealed that agreeableness might not have been ideal for assessing discriminant validity, because not all correlations between TSE and agreeableness are low. For example, Üstüner (2017) obtained a correlation of 0.37 between the same variables with PSTs in Turkey. In addition, known-group validity was well supported, with PSTs with higher TSE for both subdomains being more likely to report their professional practice being effective and a higher intention to enter the teaching profession.

## Conclusion

The present study provides evidence of the validity of a comprehensive TSE scale, in both long and short formats, tapping a broad range of teachers’ responsibilities, not limited to classroom teaching. A second-order factor structure with two subscales appears to be sufficiently established, considering the multiple EFA procedures taken, especially parallel analysis and clear factor distribution with satisfactory factor loadings, acceptable model fits for both CFAs and second-order factor analysis (after consideration is given to the effects of larger numbers of items on RMSEA; see Schermelleh-Engel et al., 2003; Matsunaga, 2010), and confirmative results in both convergent and known-group validity. Discriminant validity might not be well supported according to a rigid cutoff; however, the correlations in the present study still fall within the criteria utilized in prior TSE validation studies.

Certain implications could be drawn from the present study. First, researchers could apply the current scale either wholly, as a composite TSE score calculated as confirmation of the second-order structure, or with the subscales used separately. The teaching subscale to determine where teachers’ main interests lie for classroom teaching activities and the Ethos subscale as a measure of a broader domain of teachers’ professional development. Second, teacher educators could try to motivate PSTs to choose a teaching career through assisting them to develop a capable self-image, not limited within classroom teaching domains. Third, differentiated training shall be conducted for PSTs to enhance their TSE for Ethos, perceived as a distinctive construct than that for classroom teaching.

Certain limitations shall be taken into account when researchers interpret the results. First, only PSTs were included in the study, so the validity of this scale with ISTs is unclear. Second, the test–retest reliability of the scale has not been examined, so caution should be taken using this scale to capture changes in TSE. Third, modification indices were employed in the original two-factor and higher-order analysis by allowing errors of several items to correlate. However, the initial model fits that were less satisfactory might have been associated with a large number of items. Fourth, results on the validity were solely reported using the long format of scale considering the necessity to keep the writing consistent and condensed, despite the tests we tested with the short scale producing even satisfactory results. Therefore, future research could be directed at validating the scale with other teacher populations, including in-service teachers, in other cultural contexts, to establish its cross-cultural validity and establish the test–retest reliability of the scale in longitudinal studies. Notwithstanding, the current study provided a new comprehensive TSE scale validated using a relatively rigid procedure, including a large sample size, parallel EFA analysis, multiple reliability indicators, and validity testing with a sound theoretical assumption. Thus, we believe that this scale might serve as a good foundation for further explorations on the source of and influencing factors to teachers’ self-efficacy.

## Data availability statement

The raw data supporting the conclusions of this article will be made available by the authors, without undue reservation.

## Ethics statement

The studies involving human participants were reviewed and approved by School of Education, Yancheng Teachers University. The patients/participants provided their written informed consent to participate in this study.

## Author contributions

KM designed the research, conducted the analysis and completed the manuscript. JL methodology discussion, revised and proofread the manuscript. MC proofread the manuscript. JD collected the data and proofread the manuscript. MS revised and proofread the manuscript, and funding author. All authors contributed to the article and approved the submitted version.

## Funding

This project is supported by the National Social Science Funding for Educational Research (CHA220297) and the Beijing Normal University Start-Up Funding (no. 310432119) and Jiangsu Provincial Key Constructive Laboratory for Big Data of Psychology and Cognitive Science (206110067).

## Conflict of interest

The authors declare that the research was conducted in the absence of any commercial or financial relationships that could be construed as a potential conflict of interest.

## Publisher’s note

All claims expressed in this article are solely those of the authors and do not necessarily represent those of their affiliated organizations, or those of the publisher, the editors and the reviewers. Any product that may be evaluated in this article, or claim that may be made by its manufacturer, is not guaranteed or endorsed by the publisher.

## Footnotes

^1^ This test was conducted using the full sample (n = 984) as it was calcuated using the TSE scale items.
